# Infective endocarditis due to *Erysipelothrix rhusiopathiae* in a dog – a case report

**DOI:** 10.1186/s12917-020-02546-6

**Published:** 2020-09-10

**Authors:** Angela I. Cabrera-García, Franziska Müller, Frauke S. Rödler, Florian Traub, Romy M. Heilmann

**Affiliations:** 1 Department for Small Animals – Small Animal Internal Medicine, Veterinary Teaching Hospital, College of Veterinary Medicine, University of Leipzig, An den Tierkliniken 23, DE-04103 Leipzig, SN Germany; 2 Mobile Ultrasound Practice, Hauboldstraße 33, DE-09111 Chemnitz, SN Germany

**Keywords:** Aortic valve, *Erysipelothrix rhusiopathiae*, Infective endocarditis, Fever, Pyrexia, Zoonosis

## Abstract

**Background:**

Infective endocarditis is a rare but severe condition associated with a high mortality rate in small animal patients. This condition is caused by a microbial (most often bacterial) infection of the valvular portion of the endocardium, from which proliferative and/or erosive lesions on the cardiac valves or immediately adjacent structures develop. The two most commonly affected cardiac valves are the aortic and mitral valves.

**Case presentation:**

We report the clinical case of a 4-year old male neutered Bull terrier, 27.6 kg, body condition score 4/9, that presented with a 3-months history of pyrexia and general weakness. The patient history also revealed a transient left hind limb lameness (grade 2/4), which coincided with the onset of clinical signs about 3 months before presentation. On physical examination, a left-sided systolic heart murmur (grade 3/6) with the same intensity at the left heart base and apex, and an irregularly irregular heart rhythm were noted. Electrocardiography showed ventricular premature complexes, and echocardiography revealed lesions consistent with endocarditis involving the aortic and mitral valve. Bacterial culture of blood yielded a positive result, and the organism isolated was identified as *Erysipelothrix rhusiopathiae*. The extended patient history revealed that the dog lived close to a farm housing pigs and other livestock.

**Conclusion:**

We report a rare case of the premortal diagnosis of infective bacterial endocarditis in a dog due to *E. rhusiopathiae *infection. Most reports about this condition are from necropsy series. This clinical case report emphasizes that *E. rhusiopathiae* infection and bacteremia should be considered as a differential diagnosis in dogs with suspected infective endocarditis, especially in dogs living in rural areas with access to livestock and particularly farm pigs. Also, particular emphasis should be placed on the zoonotic potential of this infectious disease.

## Background

Infective endocarditis is a rare but very severe condition and often leads to patient death or euthanasia resulting from clinical consequences, including congestive heart failure, cardiac arrhythmias, organ infarction, sepsis, and renal failure, or a combination of these [1]. A large retrospective study including 71 canine patients showed a mortality rate of 56% in dogs with infective endocarditis, and only about half of the dogs were found to survive longer than 2 weeks in that retrospective evaluation of cases [2].

One reason for the low survival rates in canine infective endocarditis is that the microorganisms, even if isolated and appropriately treated with antimicrobials, often cannot be eliminated from the infected endocardial lesions. Also, even if complete elimination of the organism has been achieved, significant regurgitation at the level of the affected valve or valves remains due to scar tissue formation and retraction. This, in turn, can lead to circulatory volume overload and congestive heart failure. Thus, a more advanced stage of this condition at the time of diagnosis and treatment initiation generally carries a worse prognosis [3, 4].

*Erysipelothrix rhusiopathiae* is a gram-positive, non-spore-forming, facultatively anaerobic, rod-shaped bacterium that belongs to the normal microbial flora (also referred to as the microbiome) in several animal species and has a ubiquitous worldwide distribution [5]. In pigs, *E. rhusiopathiae* is the pathogen causing swine erysipelas in growing and adult swine. This disease can be associated with sudden death, pyrexia, lameness, and characteristic diamond skin lesions. Some pigs may show lethargy, cyanosis, and respiratory distress (with the risk of sudden death) due to a vegetative valvular endocarditis. There are two forms of porcine erysipelas, acute and chronic form. These two forms can occur sequentially or as a separate entity. Pigs with an acute infection are typically feverish (body temperature 40–42 °C) and hesitate to stand and move due to arthralgia and lameness. Septicemia can also occur and lead to sudden death without any prior clinical signs. The chronic form of erysipelas in swine is primarily characterized by arthritis, causing mild to severe lameness, but sudden death due to valvular endocarditis can also occur [5].

The genus *Erysipelothrix* is comprised of two species, *E. rhusiopathiae* (including serovars 1, 2, 4, 5, 6, 8, 9, 11, 12, 15, 16, 17, 19, 21 and type N) and *E. tonsillarum* (serovars 3, 7, 10, 14, 20, 22 and 23) [6]. *E. tonsillarum* can be distinguished from *E. rhusiopathiae* by serological test, the metabolic capacity of the organism to ferment saccharose, and the lack of pathogenicity in pigs [7, 8].

*E. rhusiopathiae* has been shown to cause cutaneous lesions in dogs, and affected dogs are reported to generally show nonspecific clinical signs such as lethargy, anorexia, and pyrexia [9, 10]. In humans, three different courses of illness can be caused by *Erysipelothrix rhusiopathiae* infection: a mild cutaneous infection, diffuse cutaneous lesions, or a rare systemic disease with complications including septicemia and endocarditis [5].

The following describes the clinical case of a dog with the premortal diagnosis of infective endocarditis associated with *E. rhusiopathiae* infection and bacteremia. The case documents the ability of this organism to cause infective aortic and mitral valve endocarditis in a dog.

## Case presentation

A 4-year old male neutered Bull terrier weighing 27.6 kg (body condition score 4 of 9) was presented with a 3-months’ history of generalized weakness and pyrexia (body temperature reached up to 42 °C the day before presentation), and a transient lameness (grade 2/4) noted on the left hind limb at the time of initial onset of the dog’s clinical signs. A thorough patient history revealed that the dog lived in the neighborhood of a farm housing pigs. No other relevant details could be extracted from the patient history for guiding the further diagnostic evaluation of the dog. The dog did not have a travel history outside the country of origin (Germany).

On physical examination, the dog was found to be febrile (body temperature 39.5 °C, normal body temperature in an adult dog: 38.0 – 39.0 °C). Cardiac auscultation revealed a grade 3/6 left-sided systolic heart murmur with the same intensity at the left heart base and apex and an irregularly irregular heart rhythm. No other abnormalities were noted during the physical examination. A full neurological and orthopedic examination of the dog was also within normal limits.

The minimum database comprised of a complete blood cell count and serum biochemistry revealed leukocytosis and neutrophilia, as a result of inflammation. There was a mild increase in total proteins and creatinine in blood chemistry, likely resulting from dehydration.

**Fig. 1:**
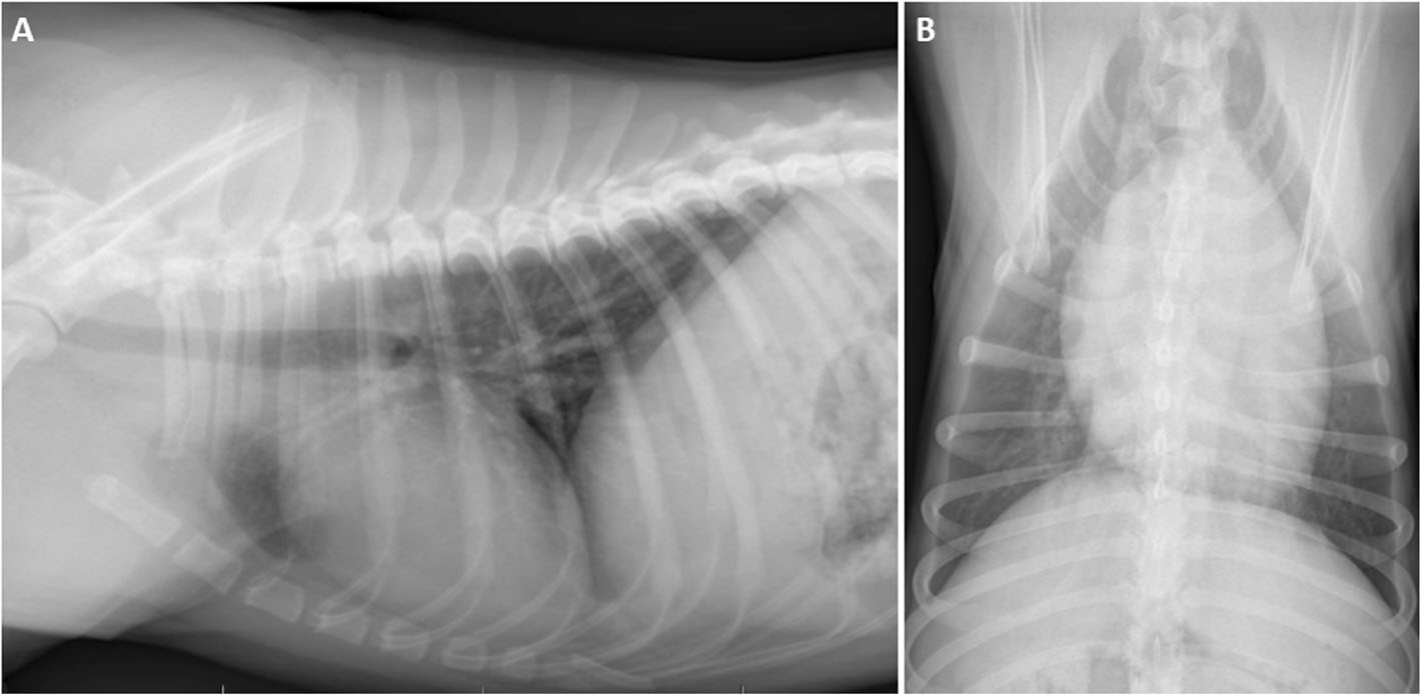
Thoracic radiographs (**a**: lateral and **b**: ventrodorsal view) at the initial presentation. An increased bronchointerstitial lung pattern (considered to be an age-related finding) and an enlarged vertebral heart score (VHS) of 11.5 (normal VHS in an adult dog: 8.5–10.5) based on which a compensated cardiomyopathy was suspected) are seen

Urinalysis showed a specific weight of 1.016 and microscopic evidence of bacteria. Urine culture was negative. Diagnostic imaging consisting of abdominal ultrasonography and thoracic radiographs was performed to further evaluate the dog for the source of a possible infection. Abdominal ultrasonography revealed no abnormalities. Thoracic radiographs (two orthogonal views) were suspicious for an underlying compensated cardiomyopathy due to an enlarged vertebral heart score (VHS) of 11.5 (reference interval: 8.5–10.5) (Fig. 1).

Arthrocentesis or joint radiographs were not performed because swelling of any joint was not noted during the physical examination, and the orthopedic examination of the dog was also unremarkable. While a monoarthropathy (e.g., infectious or traumatic monoarthritis) rarely occurs without an obvious swelling of the affected joint, mild inflammation involving multiple joints (e.g., primary or secondary immune-mediated polyarthritis) cannot be definitively excluded without synovial fluid analysis and cytological examination [11]. Additional spinal radiographs were not performed because there were no abnormal findings during the neurological and orthopedic examinations.

Further diagnostic evaluation of the dog included molecular diagnostic tests for infectious diseases (*Babesia* spp. PCR, *Anaplasma* spp. PCR, *Hepatozoon canis* PCR, and *Ehrlichia* spp. PCR) that are compatible with the clinical presentation of the dog. These tests were all negative. Also, serological evaluation for *Brucella canis* and a whole blood PCR for *Bartonella* spp. were performed, both of which were also negative.

Further evaluation of the dog to determine the source of chronic pyrexia, the heart murmur noted during cardiac auscultation, and the findings on thoracic radiographs consisted of transthoracic echocardiography and a blood culture with aerobic and anaerobic incubation. A blood culture system with a culture medium that is suitable to promote the growth of aerobic, anaerobic, and micro-aerophilic organisms (Oxoid BC0100 tryptone soya broth, gelatine peptone; Oxoid Limited, Basingstoke, Hampshire, UK) was used. Collection of blood for bacterial culture was performed longitudinally (2 h apart) and from multiple sites (three separate venipunctures: one from a cephalic vein, one from a jugular vein and one from a saphenous vein) according to current consensus, yielding six blood samples (2 ml each) altogether. Blood samples obtained from one of the 3 different venipuncture sites (3 × 2 mL) were first combined, then the total volume was added to the bottle of culture medium (2 × 3 mL). The blood samples were collected under strictly aseptic conditions using sterile disposable syringes.

**Fig. 2:**
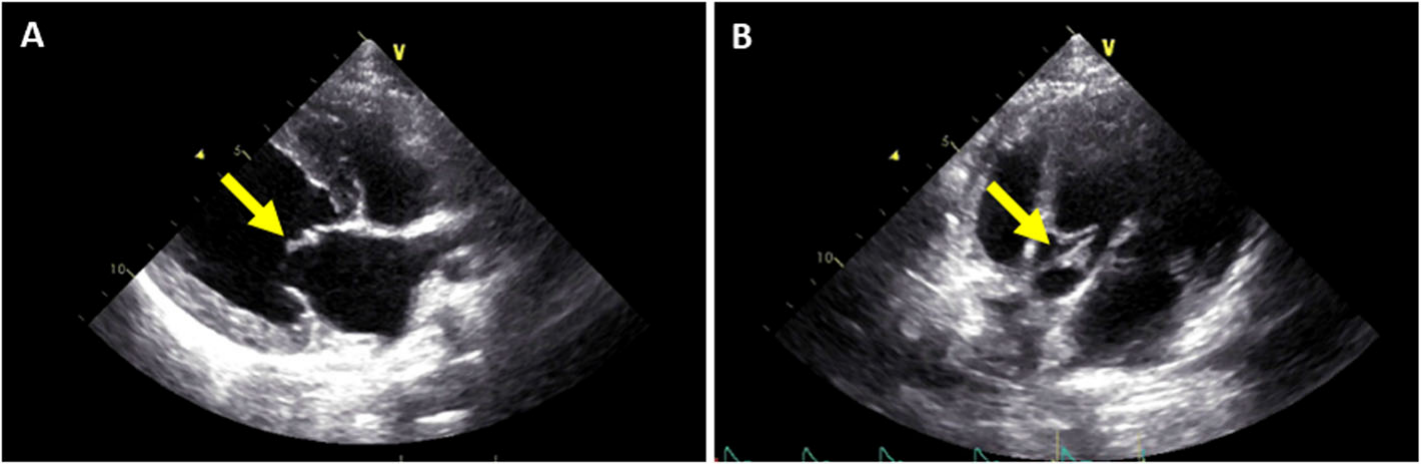
Echocardiographic view of the mitral and aortic valves. Vegetative lesions were detected **a**) on the anterior leaflet of the mitral valve (yellow arrow) in the right parasternal long-axis view and **b**) on the non-coronary cusp of the aortic valve (yellow arrows) in a left apical view optimized for evaluation of the aortic valve

Transthoracic echocardiography displayed characteristic mobile vegetations of the aortic and mitral valves (Fig. 2), with structurally altered and irregularly thickened valve leaflets (especially the aortic non-coronary cusp and the anterior mitral valve leaflet), severe aortic stenosis (max. pressure gradient: 93 mmHg), moderate aortic regurgitation (max. pressure gradient: 142 mmHg), and moderate to severe mitral regurgitation (max. pressure gradient: 212 mmHg). Pulmonic and tricuspid valves appeared structurally normal with normal pulmonary flow, but mild tricuspid regurgitation was noted. There was mild left ventricular concentric hypertrophy with normal end-diastolic and systolic dimensions. The left atrium-to-aortic root ratio (LA/Ao) was within normal limits (LA/Ao: 1.20). Ventricular extrasystoles were noted on the electrocardiogram. A presumptive diagnosis of infective endocarditis was made based on the echocardiographic findings.

Medical treatment was initiated on the day of presentation and diagnostic evaluation (day 1), while the results of aerobic and anaerobic blood culture were still pending, with enrofloxacin 5 mg/kg PO q24h (Baytril®; Bayer, Leverkusen, Germany), amoxicillin/clavulanic acid 18 mg/kg PO q12h (Synulox®; Zoetis, Parsippany, NJ, USA), sotalol 0.7 mg/kg PO q12h (Sotalol; Ratiopharm, Ulm, Germany) and clopidogrel 1.3 mg/kg PO q24h. (Plavix®; Sanofi-Aventis, Frankfurt, Germany). The dose of sotalol was reduced to 0.3 mg/kg PO q12h on day 3 because the dog developed tachycardia (noted on palpation of the chest by the owners) after sotalol administration for the first 3 consecutive doses.

One week later (day 7), the blood culture result was reported and revealed a positive result with the isolation of a gram-positive organism that was subsequently identified as *E. rhusiopathiae*. Antimicrobial susceptibility testing showed that this bacterial organism was susceptible to penicillin, amoxicillin/clavulanic acid, cephalosporine, lincomycin, fusidic acid, chloramphenicol, and metronidazole. These results led to the replacement of enrofloxacin by metronidazole 40 mg/kg PO q8h (Metrobactin® CP-Pharma, Burgdorf, Germany), whereas amoxicillin/clavulanic acid, clopidogrel and sotalol were continued as initiated. The dog’s rectal temperature decreased to normal within 72 h after switching to metronidazole, and the dog’s overall condition returned to normal.

A follow-up evaluation including echocardiography two weeks later (day 21) showed a slightly reduced aortic stenosis (pressure gradient: 82 mmHg), but the aortic and mitral regurgitation, as well as the aortic valve vegetative lesions, remained unchanged. Also, there was a severe left atrial dilation (LA/Ao: 2.03). Thus, benazepril at 0.2 mg/kg PO q24h was added to the dog’s treatment plan. Pimobendan (Vetmedin®; Boehringer, Ingelheim am Rhein, Germany) was withheld as a treatment option due to the marked aortic stenosis.

Recheck echocardiography was performed approximately every 2 weeks, and the treatment plan (as described above) was continued for approximately 3 months when the next recheck was performed (day 91). On echocardiography, aortic and mitral valve vegetations were reduced, and the left ventricular dimensions and systolic function had remained stable. However, the anterior mitral valve leaflet appeared notably immobile during diastole compared to the posterior leaflet. There was no mitral stenosis, and mitral inflow pattern was within normal limits. The left atrium remained markedly dilated.

A recheck echocardiographic evaluation of the dog two months later (day 147, delayed by 5 weeks due to owner constraints) showed the left atrial dimensions to be unchanged, but the anterior mitral valve leaflet be slightly more mobile (but not normal). Systolic dysfunction was mildly progressive, and aortic regurgitation had progressed from moderate to severe. Owner compliance was considered to be good, with reliable medication administration. Metronidazole and sotalol were discontinued, and furosemide 1.0 mg/kg PO q12h (Dimazon®; MSD, Kenilworth, NJ, USA) was added.

The dog was then lost to direct follow-up because of the emigration of the owners shortly after the last recheck examination of the dog approximately 16 weeks after initiation of medical treatment. Drug administration was continued, but no further follow-up echocardiograms were performed due to owner constraints.

The dog remained in good general and physical condition and showed no signs of disease recurrence at home (i.e., absence of pyrexia, lameness, and signs of weakness) until approximately 27 months after the initial presentation and diagnosis of infective endocarditis. At that time, the dog started to show spontaneous coughing and a moderately reduced general condition. The local veterinarian suspected pneumonia, and the owner elected humane euthanasia of the dog at the referring veterinarian due to the poor general health. A necropsy was not consented to by the owner.

## Discussion and conclusions

We report a canine case of infective endocarditis due to *E. rhusiopathiae*, the pathogen causing swine erysipelas, a very rare premortal finding in dogs.

Endocarditis infrequently occurs in small animals and is most often caused by bacterial infections. The most common bacterial organisms isolated in small animal endocarditis cases are *Staphylococcus spp.*, *Streptococcus spp.* and *Escherichia coli* [1]. *E. rhusiopathiae* is a rare cause of infective endocarditis in dogs, but most reports about this condition are post-mortem diagnoses [1, 11–13]. In contrast, *E. rhusiopathiae* is a well-known pathogen to cause endocarditis in humans and is associated with marked tissue damage to the heart valves [5]. A common pathogen that can be associated with infective endocarditis but has a high rate of false-negative blood culture results is *Bartonella* spp. [1, 2]. The aortic and mitral valves are the most commonly affected sites of infective endocarditis in dogs, and endocarditis involving the aortic valve carries a significantly worse prognosis (median survival time: 3 days) compared to dogs with endocarditis of the mitral valve (survival time 476 days) [2, 1]. Dogs with congenital aortic stenosis are predisposed to the development of infective endocarditis [1, 2].

The clinical signs of dogs with endocarditis are usually nonspecific due to the diverse sources of infections, organisms involved, and sequelae of the endocarditis [2]. The dog described here fulfilled 1 major criterion (echocardiographic findings) and 2 minor criteria (pyrexia and microbiological evidence) of the modified Duke criteria [14, 15], thus rendering infective endocarditis a possible diagnosis. The suspected arthropathy could present a third minor criterion (immunological phenomenon) and make infective endocarditis a definite diagnosis. However, the serum concentration of rheumatoid factor was not determined in this dog.

In dogs with infectious causes of endocarditis, long-term administration of antimicrobial drugs (minimum for 8–12 weeks) is usually necessary. Ideally, it should be based on the results of an antibiotic susceptibility profile following the culture and isolation of the bacterial organism. Until these results are available, dogs are usually treated with an empiric choice of an antimicrobial drug or a combination of such antimicrobial drugs (selected based on the spectrum of common causes of infective endocarditis in dogs). Preferably, antibiotics should be administered IV during the first week of treatment [1, 2].

Treating the cardiac consequences and complications of the underlying disease process, and regular patient monitoring by rechecking echocardiography and laboratory diagnostics are the most critical aspects of managing canine endocarditis cases. However, despite all treatment efforts, the progression to congestive heart failure is the most common consequence of endocarditis [1, 2].

The canine patient presented in this case report lived and roamed in the neighborhood of a farm housing pigs and other livestock. However, the *E. rhusiopathiae* status on this farm (including vaccination programs, hygiene, general biosecurity) was unknown.

Upon further probing, the owner reported that the dog had a wound on one leg approximately four weeks before the initial clinical signs (i.e., fever, weakness, and a transient lameness) developed. The affected leg was the same leg on which the dog had shown a transient lameness. A possible explanation for the lameness could be the development of a secondary immune-mediated arthritis, which as a polyarthritis, is often described as a consequence or complication of infective endocarditis and can be triggered by *E. rhusiopathiae* [10, 16]. In contrast, a septic monoarthritis appears to be an unlikely cause of this dog’s lameness because septic arthritis is highly unlikely to resolve without intervention (e.g., lavage of the affected joint). The wound had healed well, but the possibility remains that the dog roamed close to the pigs and/or other livestock during wound healing. This could have exposed the wound to the entrance of *E. rhusiopathiae* organism, which consecutively caused septicemia and infective endocarditis in this dog.

We acknowledge a few limitations of this case report. A traditional blood culture is the only possible premortal laboratory test to document the presumptive diagnosis of infectious endocarditis. However, the definitive diagnosis of infective endocarditis requires a post-mortem examination of the cardiac valves. In the case presented here, the diagnosis of infective endocarditis was based on all available premortem diagnostics (including echocardiography and result of the blood culture). However, a post-mortem pathological examination of the heart valves was not possible in this case. The cause of death in this dog also remains unclear absent the results of a post-mortem pathological examination. Pneumonia was suspected as a cause of acute worsening of clinical signs in this dog approximately 27 months after the initial diagnosis and treatment of infective endocarditis. However, the possibility of a recurrence of endocarditis or progression to heart failure and subsequent pulmonary edema cannot be ruled out.

## Data Availability

Data sharing does not apply to this article as no datasets were generated or analyzed during the current study. Further data and information about this case is available from the corresponding author upon reasonable request.

## Abbreviations

PO: Per os
LA/Ao: Left atrium-to-aortic root ratio
